# The Importance of Melanocortin Receptors and Their Agonists in Pulmonary Disease

**DOI:** 10.3389/fmed.2019.00145

**Published:** 2019-06-27

**Authors:** Anna Elizabeth Moscowitz, Huda Asif, Laurence Baily Lindenmaier, Andrew Calzadilla, Chongxu Zhang, Mehdi Mirsaeidi

**Affiliations:** ^1^School of Medicine, University of Miami, Miami, FL, United States; ^2^Division of Pulmonary and Critical Care, University of Miami, Miami, FL, United States; ^3^Section of Pulmonary, Miami VA Healthcare System, Miami, FL, United States

**Keywords:** MCR, ACTH, α-MSH, sarcoidosis, melanocortin

## Abstract

Melanocortin agonists are ancient neuropeptides that have steroidogenesis and anti-inflammatory properties. They activate melanocortin receptors (MCR), a family of five seven-transmembrane G-protein coupled receptors. MC1R and MC3R are mainly involved in immunomodulatory effects. Adrenocorticotropin hormone (ACTH) and alpha-Melanocortin stimulating hormone (α-MSH) reduce pro-inflammatory cytokines in several pulmonary inflammatory disorders including asthma, sarcoidosis, and the acute respiratory distress syndrome. They have also been shown to reduce fibrogenesis in animal models with pulmonary fibrosis. By understanding the functions of MCR in macrophages, T-helper cell type 1, and T-helper cell type 17, we may uncover the mechanism of action of melanocortin agonists in sarcoidosis. Further translational and clinical research is needed to define the role of ACTH and α-MSH in pulmonary diseases.

## Introduction

Melanocortin systems are well-known for their regulation of skin pigmentation and steroidogenesis. Interest in melanocortin receptor agonists in pulmonary medicine stems from their role as potential modifiers of inflammatory disease. In this review, we provide insight into the structures and functionality of melanocortin receptors and their agonists in pulmonary inflammatory and fibrotic diseases.

## Melanocortin Peptides

**Figure 1:**
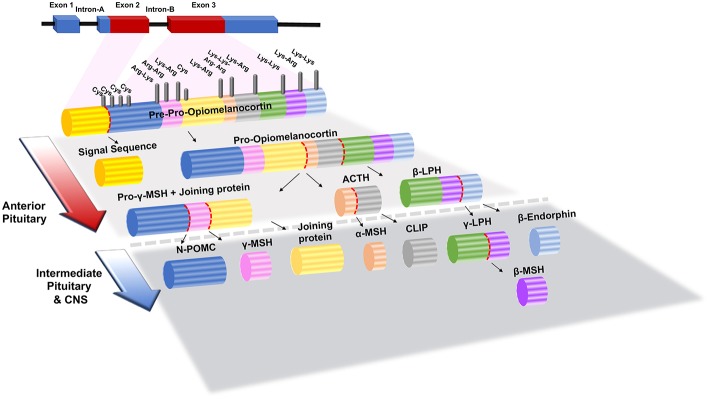
Structure of *POMC* (Pre-Opiomelanocortin) gene and its post-translational processing and modification products in the anterior and intermediate pituitary gland. Lys, Lysine; Arg, Arginine; Cys, Cystine; α-, β- and λ-MSH, α-, β- and λ-Melanocyte Stimulating Hormone; ACTH, Adrenocorticotropic Hormone; β- and λ- LPH, β- and λ- Lipotrophin; N-POMC, N-Pro-Opiomelanocortin. The figure adapted from Bicknell (1) and Mulcahy and Nillni (2) works with modification.

Melanocortin signaling peptides (melanocortins), consisting of adrenocorticotropin hormone (ACTH), α-melanocyte stimulating hormone (α-MSH), beta-melanocyte-stimulating hormone (β-MSH), and gamma-melanocyte-stimulating hormone (γ-MSH), have been studied in numerous physiologic and diseased states. All melanocortins are derived from the pro-opiomelanocortin prohormone (POMC), which is post-translationally modified by pro-convertase 1 or 2 to generate each peptide as shown in Figure 1 (3). When POMC is produced, it is uniquely cleaved in each tissue allowing ACTH/MSH-derived peptides to act locally. The melanocortin peptides contain the core amino acid sequence His-Phe-Arg-Trp, which is integral to their ability to bind their receptors (4, 5).

ACTH is maybe best known for its actions on steroidogenesis in the adrenal cortex. The adrenal cortex is divided into three histologically distinct layers: the zona glomerulosa (ZG), zona fasciculata (ZF), and zona reticularis (ZR). The ZG is predominantly responsible for secretion of aldosterone which is primarily regulated by renin. The ZF and ZG secrete glucocorticoids and the androgen precursors, dehydroepiandrosterone (DHEA), respectively, both of which are controlled by ACTH secretion (6). While the bulk of research has been on the adrenal actions of ACTH, recent studies have begun to shed light on extra-adrenal functions. Those studies have focused on the influence of adipocyte differentiation and function as well as matrix synthesis in mesenchymal cells (7–9). It has been proposed that ACTH increases intracellular calcium in mesenchymal cells and initiate differentiations. (10) Other roles of ACTH that are beginning to be explored are thymic cell growth and differentiation, androgen production via direct action on Leydig cells, a reno-protective effect in chronic kidney disease, and influences on mood (6).

α-MSH is a 13 amino acid peptide most known for its cutaneous response to ultraviolet light leading to increased skin pigmentation (11). It has also been shown to possess anti-inflammatory and anti-microbial effects all via melanocortin receptor signaling (12–14). β-MSH and γ-MSH have been studied considerably less than α-MSH or ACTH. β-MSH does not exist in rodents due to lack of N-terminal cleavage site (15). Therefore, there is little information on its functionality. Recently, a mutation in β*-MSH* gene (Y5C-β-MSH) was associated with obesity in children and highlighted the essential role of β-MSH in the hypothalamic control of body weight in humans (16).

γ-MSH was named for homology to alpha and beta but without known melanotropic activity (17). γ-MSH has 2 cleavage products gamma-1 and gamma-3, both with a critical N-terminal lysine residue. The work that has been done on Lys-gamma3-MSH has shown that it functions both *in vitro* and *in vivo* to potentiate the steroidogenic effects of ACTH on the rat adrenal. Specifically, it was demonstrated that they act together to increase the activity of hormone sensitive lipase (18).

## Melanocortin Receptors

**Figure 2:**
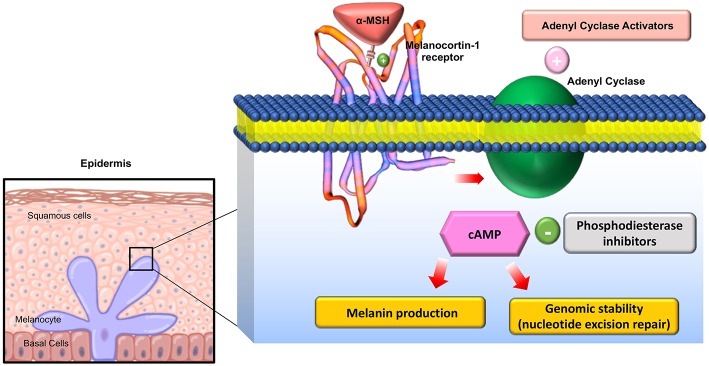
Downstream effects of Melanocortin-1 receptor stimulation in melanocytes: On stimulation of the receptor, Adenyl cyclase is activated and cAMP is generated activating multiple downstream mediators involved in melanogenesis and melanocyte proliferation. Adapted from Joshi et al. (22) work with modification.

**Figure 3:**
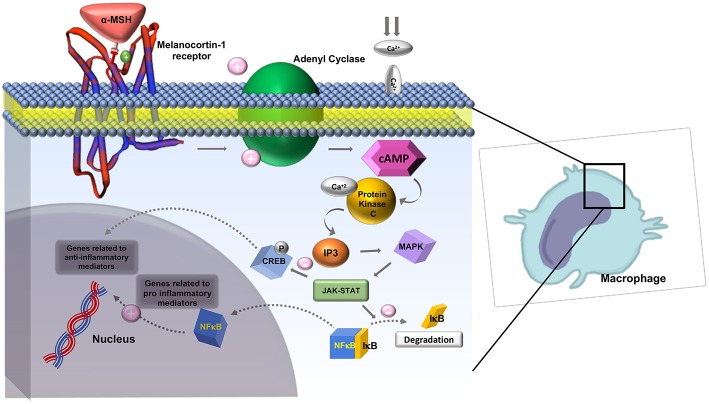
Role of Melanocortin-1 receptor on macrophages in inhibiting inflammation. MC1R activates adenyl cyclase and generates cAMP, activating protein kinase-C. This leads to influx of extracellular calcium and activation IP3. IP3 then activates MAPK and JAK-STAT pathways which inhibit the degradation of IκB and activate CREB. CREB is involved in downstream anti-inflammatory effects. Adapted from Zhang et al. (23) work with modification.

The melanocortin receptors are a family of five seven-transmembrane G-protein coupled receptors (GPCRs) and are the smallest GPCRs known. Their transmembrane domains are alpha-helical with the conserved motif of aspartic acid-arginine-tyrosine at the junction of the TM3 domain and contain cysteine at the C terminus (19). Human melanocortin receptor genes are located on single exons within autosomes. In humans, MC1R is found on chromosome 16, MC3R is on chromosome 20, while MC2R, MC4R, and MC5R are on chromosome 18 (20). Sequence homologies among the receptors range from 38 to 60%, with MC2R demonstrating the least homology with the other receptors (21). Each receptor is coupled to adenylate cyclase, triggering formation of cAMP and activating protein kinase C. This leads to influx of extracellular calcium resulting in IP3 activation. IP3 then activates the MAPK, and JAK-STAT pathways (as shown in Figures 2, 3) (23). The receptors are widespread throughout the body, exhibiting myriad ligand affinities, tissue and cell distribution, and downstream effects (24).

**Figure 4:**
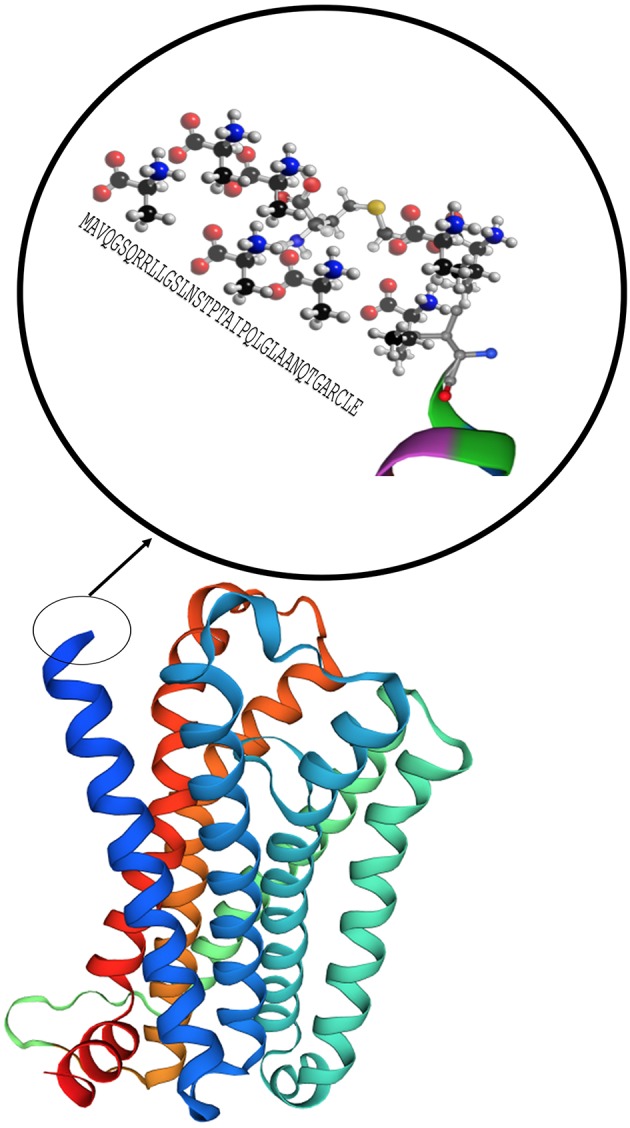
Model of the MC1R secondary structure with 7 core domains and a rich tail. Modeling was performed by using Swiss-Model (26).

MC1R was first cloned in 1992 and has been studied extensively for its role in regulation of human integumentary pigmentation. It contains 317 amino acids and was originally referred to as α-MSH receptor, the name of its major ligand (25). MC1R is formed from 7 trans-cytoplasmic membrane segments and an extramembrane domain contains 37 AA as shown in magnification circle in Figure 4 (27). Video 1 shows a 3D secondary molecular structure of MC1R. MC1R has equal affinity for α-MSH, ACTH, and β-MSH, but less to γ-MSH (24). MC1R on human melanocytes is stimulated by its ligands to enhance melanocyte proliferation and melanogenesis as shown in Figure 2. This receptor is highly polymorphic and its variants have been associated with increasing risk of melanoma and non-melanoma skin cancers (22). MC1R is expressed on endothelial cells, monocytes, macrophages, lymphocytes, neutrophils, mast cells, intestinal epithelia, as well as, in testicular, ovarian, placental, lung, and liver tissue (28). Mutations in the *MC1R* gene may modify the immune response in the setting of inflammation. Seaton and co-workers reviewed 1,246 subjects with blunt injury and found that those who carried a polymorphism at the rs885479 location of *MC1R* gene had a lower risk (OR = 0.45, *P* = 0.006) of complicated sepsis (29).

The *MC2R* gene is located on chromosome 18 and contains 325 amino acids (30). MC2R is predominantly expressed in the adrenal cortex, where it mediates the effects of ACTH. It is believed to bind ACTH exclusively, which is crucial in regulating steroidogenesis (31). Mutations affecting the normal function of MC2R are implicated in familial glucocorticoid deficiency, including polymorphism on S74I, R146H/560delT, and 579-581delTGT (32).

MC3R, a 361 amino acids-long peptide coded on chromosome 20, is the least selective of the melanocortin receptors, binding the four melanocortins with equal affinity (33). MC3R expresses on macrophages and intestinal epithelial cells. It is also found in lymphocytes, brain, heart, and placental tissues, as well as skeletal muscles (28, 34).

Human MC4R was cloned by Gantz et al. (33), who traced it to chromosome 18 as a gene encoding a protein with 333 amino acids in length (35). MC4R is expressed throughout the central nervous system (36). It has similar affinity to ACTH, α-MSH, and β-MSH, and to a much lesser extent to γ-MSH. MC4R is involved with energy homeostasis, sexual behavior, nociception, inflammation, and neuroprotection (37, 38). Inhibition of MC4R in mice results in obesity secondary to a hyperphagic, hyperinsulinemic, and hyperglcyemic state (39).

MC5R was the last of the melanocortin gene family to be cloned by homology screening from human genomic DNA in 1993 and subsequently in the mouse genome the following year (40).

The MC5R has a similar ligand recognition affinity as MC1R and MC4R in which it binds most avidly to α-MSH and to a lesser extent ACTH. It does not bind to γ-MSH (41). MC5R expresses in numerous tissue types including the adrenal glands, fat cells, as well as kidney, thymus, skin, testicular, ovarian, uterine, esophageal, duodenal, liver, and lung tissue. It is also found in lymph nodes, bone marrow, skeletal muscle, and exocrine glands (42). The presence of MC5R on both B- and T- lymphocytes suggests it may play a role in regulation of the immune system and inflammatory response (42). MC5R has a role in exocrine gland function and thermoregulation. In the MC5R knockout mouse model, water repulsion and thermoregulation are impaired as a result of decreased production of sebaceous lipids (43). Recently, Trotta et al. proposed that stimulation of MC5R with α-MSH reduced risk of high glucose-induced cardiac hypertrophy (44).

## Association of Melanocortin Receptors to Inflammatory Diseases

MC1R and MC3R are the most studied members of the melanocortin system family with respect to inflammation. They may be potentially significant in the context of pulmonary diseases. Evidence of the anti-inflammatory properties of the melanocortin system are described in dermatologic, central nervous system, and pulmonary disorders.

## Dermatitis

Chen et al. investigated the immunoregulatory effects of MC1R. They found that MC1R inhibited the inflammatory response in Raw 264.7 cells and atopic dermatitis (AD) mice model (45). AD is a chronic allergic disease with severe irritation and inflammation of the skin (46). An AD model in mice was developed by using contact allergens. It was shown that α-MSH suppressed dermatitis in these AD mice (45). Interestingly, it has been shown that the agonists of MC1R and MC5R have inhibitory effects on IgE-mediated allergic inflammation (46).

## Fever and CNS Inflammation

Early into the investigation of α-MSH, it was shown that it acted as an antipyretic in the setting of experimental fever (47). Glyn and Lipton induced fever by injection of either endogenous or exogenous pyrogens. The pyogenic response was significantly reduced regardless of bacterial endotoxin or PGE2 injection (47). Their findings were later validated by others in rabbit and monkey models (48, 49).

Han et al. studied the effect of α-MSH on nerve repair. They developed a mice autoimmune encephalitis model and suggested that α-MSH promoted the restoration of injured nerves on the spinal cord. Furthermore, α-MSH has an inhibitory effect on the secretion of pro-inflammatory cytokines including IL-2 and IFN-γ (50).

## Respiratory Inflammatory Diseases

The presence of MC1R and MC3R were detected on wild type mice alveolar macrophages (51). Getting et al. showed that melanocortin peptides inhibit leukocyte accumulation in a mice model of pulmonary allergic and non-allergic inflammation. They found that this protective effect is associated with the activation of MC3R on alveolar macrophages (51).

Allergic airway disease is characterized by exaggerated airway response to agents in the environment. Raap et al. developed a mice model for asthma by three intraperitoneal injections and inhalations of 10 μg of ovalbumin (52). To understand the role of melanocortin signaling in asthma, they injected 1 mg/kg of α-MSH into the tail of mice before sensitization. They found a significant reduction in eosinophil counts in bronchoalveolar lavage (BAL) (*P* < 0.001) and a significant decrease in serum levels of IgE (*p* < 0.001) IL-4 and IL-5 (both *p* < 0.001) among asthmatic mice in comparison to controls. Their study suggests melanocortin receptor agonists have anti-Th2 T-cells activity.

Colombo et al. recently conducted an animal study to discover the potential therapeutic effects of α-MSH on acute lung injury (ALI). They developed an ALI rat model by injecting a 1 mg bleomycin instillation into the trachea. They injected 100 μg of α-MSH immediately after bleomycin and harvested lungs 8, and 24 h later in case and control groups. They found α-MSH significantly prevented lung edema (lung weight in control vs. α-MSH group, 5.8 ± 0.5 vs. 3.9 ± 0.1, respectively, p <0.05 respectively), and reduced circulatory NO (nitrite) concentrations in the case group. Several genes associated with inflammation including Cdkn1a, Hmox1, and Hspa1a were upregulated in mice with ALI that had normalized in the mice injected with α-MSH. Their findings support potential therapeutic properties of α-MSH in ALI (53).

Jiangping et al. discovered the therapeutic effects of α-MSH in ALI by using a mouse model of ALI. They caused bilateral renal ischemia for 40 min in mice to develop ALI. The mice were then given 25 mg of α-MSH or saline in case and control groups before the clamps were removed. The treatment with α-MSH was repeated at 8 and 16 h post ischemia. The leukocyte numbers and injury score in lung specimens demonstrated significant reduction in the α-MSH group in comparison with saline control group. The α-MSH group had significantly lower expressions of *TNF-*α and intracellular adhesion moelcul-1 (*ICAM-1*) genes after renal ischemia (54).

Kristensen et al. investigated the role of α-MSH in the systemic inflammatory response syndrome (SIRS). This study employed the use of AP214 which is an analog of α-MSH with six lysine residues at the N-terminus of native α-MSH, resulting in a higher binding affinity to its receptors. They developed a porcine model for SIRS with intravascular infusion of lipopolysaccharide (LPS from Escherichia coli endotoxin). Animals were then randomized to receive either AP214 or saline injections. When the porcine is exposed to LPS, it shows a dose dependent increase in pulmonary vascular resistance, a well-known pathophysiologic change in acute respiratory distress syndrome (ARDS) which is also observed in sepsis. ARDS in the setting of sepsis is triggered in part by endothelial damage as a result of the inflammatory process. Remarkably, the peak pulmonary pressures and pulmonary vascular resistance were significantly (~33%) reduced in the AP214 group (55).

The mechanism of action of α-MSH to inhibit inflammation is not well-known. It is suggested that α-MSH exhibits its effect in attenuating the inflammatory response by down regulating nuclear factor kappa B (NFκB) (56). NFκB is a nuclear transcription factor that plays a key role in the cytokine production. In the absence of inflammatory signals, NFκB is inactive as a heterodimeric molecule, which is composed mainly of two subunits, p65 (RelA), and p50 (NF-κB_4_) (57). Furthermore, a regulatory protein of NFκB, IκB acts as a molecular off switch. When inflammatory signals are present, IκB undergoes phosphorylation, ubiquitination, and proteolytic degradation allowing for the activation of NFκB, in particular the p65 subunit (57). Authors believe that the cAMP response element binding protein (CREB) activation has a central role in reducing levels of a few pro-inflammatory cytokines such as IFN- γ and IL-7 as shown in Figure 2.

## Organ Fibrosis

Fibrotic and sclerotic conditions consist of a heterogeneous group of disorders including hypertrophic scars, keloids, scleroderma, systemic sclerosis, cirrhosis of the liver, and interstitial lung fibrosis.

Extracellular matrix (ECM) remodeling is the common denominator in every fibrotic and sclerotic disease regardless of etiology (58). One of the most common models to study fibrosis is the bleomycin model (BLM). Kokot et al. developed a mice model of scleroderma by subcutaneously injecting bleomycin 10 μg per day for 21 days. Mice were treated with α-MSH (5 μg subcutaneously per day) for 3 weeks. Treatment with α-MSH suppressed BLM-induced expression of type I and type III collagen an effect which was cAMP-dependent (59). Luo et al. recently conducted a study asking whether melanocortin signaling is altered in keloid formation. Normal and keloid human fibroblasts were cultured to compare melanocortin receptor expression. In keloid-derived fibroblasts, *MC1R* gene expression was significantly reduced by less than half compared to normal fibroblasts using RT-PCR (60).

Liver fibrosis results from a progressive accumulation of fibrillar ECM in the liver as a consequence of repeated liver damage. Lee and co-workers developed a liver fibrosis model with injection of carbon tetrachloride (CCl_4_) 1 ml/kg body weight twice a week for 10 weeks (61). Mice were electrotransferred with the *ACTH* 1-17 gene after fibrosis development. Histopathology and measurement of collagen contents of the livers showed that transfected mice significantly reversed CCL_4_ liver fibrosis in comparison to controls (liver collagen content in α-MSH group was 23.7 ± 4.7 vs. 59.7 ± 5.0 μm/mg in control group, *p* < 0.01).

The effects of α-MSH on attenuating fibrosis is due to a reduction in pro-inflammatory cytokine production and attenuation of procollagen synthesis (62).

Xu et al. investigated effects of an α-MSH analog on a bleomycin pulmonary fibrosis mice model. They found that an α-MSH analog reduced the mRNA expression of type I and III procollagen and production of hydroxyproline in the bleomycin treated mice. They also demonstrated that lung production of TNF-α, IL-6, macrophage inflammatory protein 2, and TGF-β1 was significantly reduced in the α-MSH analog treated group in comparison to controls (63).

## Melanocortins and Sarcoidosis

Sarcoidosis is a complex, multisystem disorder characterized by non-caseating granulomatous inflammation (64). The presentation of sarcoidosis ranges greatly from asymptomatic with incidental findings on imaging, to significant morbidity and potential mortality secondary to mainly pulmonary insufficiency and cardiac arrhythmia (65). Though there are myriad manifestations of the disease, the lungs and thoracic lymph nodes are almost always involved, causing cough, dyspnea, or a decline in lung function (66). Immunosuppression is the mainstay of treatment as the disease is believed to be mediated largely via immune system dysregulation (67).

Prednisone and ACTH with 39 amino acid (repository corticotropin, a long acting corticotropin) are the only two medications currently approved by The Food and Drug Administration (FDA) for the treatment of sarcoidosis (68, 69). ACTH binds with MCRs and attenuates pro-inflammatory cytokines that play a central role in sarcoidosis. Baughman et al. used high dose repository corticotropin (80 IU) twice per week for 47 subjects with advanced sarcoidosis (70). Twenty seven out of 29 subjects who finished 6 months of treatment showed improvement or stability of disease. Reduction of the oral corticosteroid dose was recorded in 27 subjects. Eighteen subjects were treated for <3 months due to side effects, death, or cost. The observed effect of repository corticotropin in this study could be beyond the effect of steroidogenesis in the adrenal glands. Repository corticotropin may also impart anti-inflammatory properties on immune cells via MCR agonist effects (71).

It has been shown that the granulomatous inflammation of sarcoidosis is regulated by T helper 1 (Th1) activating cytokines including IFN-γ, interleukin-12. There is also a decreased expression of the T helper 2 (Th2) cytokines IL-4 and IL-5 (72). Of note, IL-12 has been shown to be the key regulator of the Th1 immune response and is upregulated in lungs of sarcoidosis. It has been shown that α-MSH inhibits the release of pro-inflammatory Th1 cytokines from alveolar macrophages. When macrophages are treated with an inflammatory endotoxin, there is increased secretion of IL-12 and IFN-γ compared to controls. When these alveolar macrophages are exposed to α-MSH, the production of those Th1 cytokines is significantly decreased (73).

## Future Perspectives

Although α-MSH has been studied for decades, the field of melanocortin agonists in pulmonary disease is in its infancy. The new studies, particularly those targeting the effects of melanocortin agonists in pulmonary diseases should be integrated with genomics and proteomics to gain a complete picture of biologic and cellular processes. The role of MC1R on the lung immune system should be studied more. The effect of MC1R agonists including a-MSH may improve inflammatory lung diseases including sarcoidosis. Our team developed a set of experiments to study the anti-inflammatory properties of α-MSH in sarcoidosis by using an *in-vitro* and mice model.

Validation via large multicentric studies should be done to confirm the therapeutic properties of melanocortin agonists in sarcoidosis, ILD, and airway diseases.

## Author Contributions

AM and LL conducted literature review, conducted exploratory analysis and helped to develop the first draft of the manuscript. HA helped to develop figures of the manuscript. AC conducted literature review, and helped in developing the first draft of the manuscript. CZ conducted literature review and helped in writing the paper. MM conducted literature review, conducted exploratory analysis, modeled the 2nd protein structure, and developed the final version of the manuscript.

### Conflict of Interest Statement

The authors declare that the research was conducted in the absence of any commercial or financial relationships that could be construed as a potential conflict of interest.

## Supplementary Material

Video 1 The 3rd structure of MC1R by using Swiss-Model (26).
